# Angiotensin converting enzyme 2 in patients with sepsis associate with comorbidities but neither with mortality nor with organ failure

**DOI:** 10.1038/s41598-025-96640-0

**Published:** 2025-04-23

**Authors:** Felix Forsberg, Toralph Ruge, Anders Larsson, Per Wändell, Axel C. Carlsson, Peter M. Nilsson, Per Swärd

**Affiliations:** 1 Clinical and Molecular Osteoporosis Research Unit, Departments of Orthopedics and Clinical Sciences, Skåne University Hospital, Lund University, 205 02 Malmö, Sweden; 2 Department of Clinical Sciences Malmö & Department of Internal Medicine, Skåne University Hospital, Lund University, Malmö, Sweden; 3 Department of Emergency and Internal Medicine, Skånes University Hospital, Malmö, Sweden; 4 Department of Medical Sciences, Uppsala University, 751 85 Uppsala, Sweden; 5 Department of Neurobiology, Care Sciences and Society, Karolinska Institutet, Alfred Nobels Allé 23, 141 83 Huddinge, Sweden; 6 Department of Clinical Sciences, Skåne University Hospital, Lund University, 205 02 Malmö, Sweden

**Keywords:** Angiotensin converting enzyme 2 (ACE2), Comorbidities, Level of care, Mortality, Organ failure, Sepsis, Bacterial infection, Cardiovascular biology

## Abstract

High levels of circulating angiotensin converting enzyme 2 (ACE2) are associated with several chronic diseases and mortality risk. Less is known about the prognostic value of ACE2 in patients with sepsis. In the present study, we aimed to investigate the association between plasma ACE2 levels on admission to the ED and 28-day mortality, organ failure, and level of care in a prospectively recruited observational study sample. Six hundred patients with sepsis admitted to the emergency in Malmö 2013–2015 were included in the analysis. Uni- and multivariable binary logistic regression was conducted to investigate the association between ACE2 and 28-day mortality, organ failure, and level of care. Plasma ACE2 levels were increased in patients with male sex, high age, and comorbidities, including diabetes, cardiovascular disease, and cancer. Plasma ACE2 levels associated with hospitalization and intermediate care unit stay in univariate but not multivariate analysis. Plasma ACE2 did neither associate with 28-day mortality, OR 1.19 (95% CI 0.86–1.65, *p* = 0.29), nor with organ failure, OR 1.08 (95% CI 0.73–﻿1.56. p = ﻿0.72). Future studies investigating the dynamics of circulating ACE2 levels in patients with sepsis longitudinally are warranted.

## Introduction

Angiotensin converting enzyme 2 (ACE2) is an enzyme catalyzing the hydrolysis of angiotensin (ANG) II into ANG 1–7, as a counter-regulatory mechanism of the renin-angiotensin system (RAS)—i.e. alternative RAS signaling, as opposed to classical RAS activity, promoting ANGII signaling^1^. ACE2 is attached to cell membranes (mACE2) in several organ systems of the body. The expression is high in intestines, testis, ovaries, kidneys, adipose tissue, cardiovascular tissue, mammary tissue, and the thyroid, but it also appears at lower quantities in the lungs, the nervous system, liver and other tissues^2^ [https://gtexportal.org/home/gene/ACE2]. The sheddase (enzyme) a disintigrin and metalloproteinase 17 (ADAM17) is believed to be important in cleaving the transmembrane domain of mACE2, especially under inflammatory conditions, making it soluble (sACE2)^3,4^.

ANG 1–7 acts as an anti-inflammatory biomarker and decreases blood pressure through vasodilatation, diuresis, and natriuresis. ANG II on the other hand is pro-inflammatory and increases blood pressure through vasoconstriction, sympathetic activity, renal water/sodium reabsorption and stimulation of aldosterone secretion. ACE2 favors decrease in blood pressure and acts anti-inflammatory as it decreases ANG II and increases ANG 1–7 levels^5,6^.

During the global coronavirus disease (Covid-19) pandemic 2019–2022, mACE2 has been identified as the receptor and main cell entry point for the severe acute respiratory syndrome coronavirus 2 (SARS-CoV-2)^7,8^. Studies have found that high plasma ACE2 levels predict severe COVID-19, and that ACE2 levels increased over time from the first arrival of patients to the emergency department (ED)^9,10^. Studies also show that plasma ACE2 is a biomarker of adverse cardiovascular events and mortality in the general population^11^. Less is known about the predictive value of high plasma ACE2 levels in acute severe manifestations of infectious diseases other than COVID-19, including sepsis. One study reported that children with sepsis-associated organ dysfunction had higher plasma ACE2-levels than children with sepsis alone^12^. Recently, studies have shown that ACE2 can protect against both cardiac dysfunction, vascular dysfunction, septic hypotension and neuroinflammation in sepsis-induced mice^13–15^.

We hypothesized that plasma ACE2 is associated with the degree of sepsis severity and that circulating ACE2 levels are associated with mortality, organ failure, and level of care. The *aim* of this observational study was to study the association between ACE2 levels on admission to the ED and 28-day mortality, organ failure, and level of care in a prospectively recruited observational study sample.

## Results

Of 610 available subjects, 10 subjects were excluded. Seven subjects were excluded due to missing ACE2 values, and three subjects were excluded due to missing data regarding survival or not, leaving 600 subjects included in the analysis. Of these 600 subjects, 55 (9.2%) did not survive the first 28 days after admission to the emergency department. Patient characteristics are displayed in Table 1.

**Table 1:** Characteristics and outcome of the population with comparison between 28-day survivors and non-survivors.

Baseline Characteristics	Sepsis cohort	Non-survivors 28d	Survivors 28d	*p* value
Number, n (% of sepsis cohort)	600	55 (9.2)	545 (90.8)	
Age in years, median (IQR)	73 (61–82)	80 (73–88)	72 (59–82)	< 0.001
Female sex, n (%)	292 (48.7)	25 (45.5)	267 (49.0)	0.617
Body mass index (MV = 25), median (IQR)	25.8 (22.5–29.8)	25.2 (22.1–28.5)	25.8 (22.5–30.0)	0.26
**Comorbidities**				
Cardiovascular disease (MV = 2), n (%)	182 (30.4)	27 (49.1)	155 (28.5)	0.002
Respiratory disease (MV = 1), n (%)	140 (23.4)	19 (34.5)	121 (22.2)	0.040
Neurological disease (MV = 3), n (%)	108 (18.1)	9 (16.7)	99 (18.2)	0.776
Renal disease (MV = 6), n (%)	45 (7.5)	5 (9.3)	40 (7.4)	0.590
Cancer (MV = 3), n (%)	164 (27.4)	20 (37.0)	144 (25.5)	0.099
Immunodeficiency (MV = 9), n (%)	32 (5.4)	6 (11.1)	26 (4.8)	0.061
Diabetes (MV = 1), n (%)	114 (19.0)	14 (25.5)	100 (18.4)	0.203
Psychiatric disorder (MV = 3), n (%)	50 (8.4)	4 (7.4)	46 (8.5)	1.000
Limitation of care (MV = 5), n (%)	91 (15.3)	24 (43.6)	67 (12.4)	< 0.001
Use of ACE-I or ARB (MV = 6), n (%)	149 (25.1)	14 (25.5)	135 (25.0)	0.947
**Site of infection (MV = 58)**				
Pulmonary, n (%)	197 (36.3)	23 (53.5)	174 (34.9)	0.137
URTI, n (%)	51 (9.4)	0 (0)	51 (10.2)	0.010
Urinary, n (%)	132 (24.4)	4 (9.3)	128 (25.7)	0.017
Bone and joint, n (%)	7 (1.3)	1 (2.3)	6 (1.2)	0.492
SSTI, n (%)	58 (10.7)	7 (16.3)	51 (10.2)	0.420
Gastrointestinal, n (%)	22 (4.1)	1 (2.3)	21 (4.2)	0.711
Other, n (%)	75 (13.8)	7 (16.3)	68 (13.6)	0.957
**Outcomes**				
No organ failure, n (%)	285 (47.5)	13 (23.6)	272 (49.9)	< 0.001
Intermediate organ failure (1–3), n (%)	282 (47.0)	30 (54.5)	252 (46.2)	0.006
Severe MOF (≥ 4), n (%)	33 (5.5)	12 (21.8)	21 (3.9)	< 0.001
ICU admission (MV = 3), n (%)	28 (4.7)	7 (12.7)	21 (3.9)	< 0.001
Discharged from ED (MV = 2), n (%)	68 (11.4)	2 (3.6)	66 (12.2)	0.058
**Biomarkers**				
ACE-2 ng/mL, median (IQR)	4.6 (2.7–9.4)	5.0 (3.1–10.2)	4.6 (2.64–9.29)	0.27
Lactate (MV = 23) mmol/L, median (IQR)	1.7 (1.3–2.7)	2.1 (1.3–3.1)	1.7 (1.3–2.7)	0.08
CRP (MV = 7) mg/L, median (IQR)	73.0 (25.0–163.0)	106.5 (47.5–185.0)	70.0 (24.0–160.0)	0.04
Creatinine (MV = 5) µmol/L, median (IQR)	88.0 (68.0–119.0)	100.0 (77.3–157.3)	86.0 (68.0–117.0)	0.02
**Vital signs**				
Respiratory rate (MV = 4)	28 (22–32)	30 (22–36)	27 (22–32)	0.096
Oxygen saturation (MV = 3)	93 (88–96)	90 (85–93)	93 (88–97)	< 0.001
Heart rate (MV = 1)	106 (94–120)	100 (90–115)	106 (95–120)	0.11
Systolic blood pressure (MV = 5)	137 (120–150)	120 (107.5–149.5)	138 (120–151)	0.01
Diastolic blood pressure (MV = 84)	77 (66–86)	70 (60–80.5)	77 (67–86)	0.007
Mean arterial pressure (MV = 84)	97 (95–98)	91(85–97)	97 (96–99)	0.06
Body temperature (MV = 0)	38.8 (38.3–39.3)	38.6 (38.0–39.2)	38.8 (38.3–39.3)	0.13

The *P* value shows the difference between survivors and non-survivors. The percentage (%) is calculated within the respective subgroup.

*MV* Missing values, IQR interquartile range, *ACE-i* angiotensin convertin enzyme inhibitor, *ARB* Angiotensin II receptor blocker, *URTI* upper respiratory tract infection, *SSTI* skin and soft tissue infection, *MOF* multiple organ failure, *ICU* intensive care unit, *ED* emergency department, *ACE2* angiotensin converting enzyme 2, *CRP* C-reactive protein. Respiratory rate is counted as breaths per minute, oxygen saturation is in % saturation received from pulse oximeter, heart rate is counted in beats per minute, systolic, diastolic and mean arterial blood pressure are all counted in mmHg, body temperature is counted in degrees Celsius.

Cardiovascular disease, respiratory disease, organ failure, and decision on limitation of care were attributes that showed a significantly increased risk of mortality within 28 days (*p* = 0.002, *p* = 0.040, *p* < 0.001, *p* < 0.001, respectively). Patients with upper respiratory tract infection (URTI) or urinary infection (UTI) had a lower 28-day mortality (*p* = 0.010, *p* = 0.017, respectively).

Plasma ACE2 levels ranged between 0.46 and 239.86 ng/ml in the whole study population.

Looking at tendencies in plasma ACE2 levels, certain comorbidities and attributes were associated with plasma ACE2 levels when compared to their respective counterparts. Age, male sex, obesity, cardiovascular disease, cancer and diabetes mellitus were all associated with higher plasma ACE2 levels (Table 2).

**Table 2:** Comparison of plasma ACE2 levels in subgroups.

Differences in ACE2	n (%)	Yes	No	*P* Value
Non-survivors 28d (MV = 0)	55 (9.2)	4.99 (3.08–10.20)	4.60 (2.64–9.29)	0.268
Age > 70 (MV = 0)	241 (40.2)	5.45 (3.04–10.20)	3.93 (2.64–9.29)	< 0.001
Female sex (MV = 0)	292 (48.7)	4.30 (2.35–8.46)	5.23 (3.05–10.16)	0.010
Obesity (MV = 25)	141 (24.5)	6.45 (3.25–11.60)	4.30 (2.44–8.96)	< 0.001
Cardiovascular disease (MV = 2)	182 (30.4)	5.52 (3.06–9.94)	4.30 (2.33–9.08)	0.014
Respiratory disease (MV = 1)	140 (23.4)	4.63 (2.66–8.32)	4.64 (2.65–9.51)	0.875
Neurological disease (MV = 3)	108 (18.1)	4.76 (2.84–10.07)	4.54 (2.64–9.29)	0.473
Renal disease (MV = 6)	45 (7.6)	6.26 (3.43–10.56)	4.51 (2.65–9.10)	0.130
Cancer (MV = 3)	164 (27.5)	5.47 (3.12–10.28)	4.39 (2.56–8.80)	0.027
Immunodeficiency (MV = 9)	32 (5.4)	5.24 (3.08–11.44)	4.51 (2.63–8.90)	0.411
Diabetes mellitus (MV = 1)	114 (19.0)	6.58 (3.54–12.76)	4.30 (2.58–8.53)	< 0.001
Psychiatric disorder (MV = 4)	50 (8.4)	4.68 (2.70–9.87)	4.64 (2.65–9.28)	0.720
Limitation of care (MV = 5)	91 (15.3	4.96 (3.07–9.94)	4.48 (2.60–9.30)	0.191
Hypertension (MV = 2)	244 (40.8)	6.15 (3.25–10.99)	4.06 (2.24–7.99)	< 0.001
No organ failure (MV = 0)	285 (47.5)	4.30 (2.42–9.17)	4.89 (2.84–9.68)	0.179
Intermediate organ failure (MV = 0)	282 (49.7)	4.85 (2.78–9.82)	4.30 (2.42–9.17)	0.189
Severe MOF (MV = 0)	33 (5.5)	4.93 (3.03–8.24)	4.60 (2.65–9.41)	0.799
ICU admission (MV = 3)	28 (4.7)	4.23 (2.18–9.36)	4.64 (2.69–9.36)	0.378
Discharged from ED (MV = 2)	68 (11.4)	3.42 (2.09–5.87)	4.85 (2.77–9.94)	0.001

Data showing the difference of plasma ACE2 levels between patients with and without certain attributes and outcomes.

*MV* missing values, *MOF* multiple organ failure, *ICU* intensive care unit, *ED* emergency department, *ACE2* angiotensin converting enzyme 2.

### Association between ACE2 levels and 28-day mortality

In neither the univariate nor the multivariate binary logistic regression analysis any significant association between plasma ACE2-levels and 28-day mortality could be found. The OR in the univariate and the multivariate were 1.17 (95% CI 0.90–1.54, *p* = 0.24) and OR 1.19 (95% CI 0.86–1.65, *p* = 0.29) respectively (Table 3).

**Table 3:** Uni- and multivariable binary logistic regressions showing what impact plasma ACE2 levels have on outcome per standard deviation, presented as odds ratios.

Univariate				Multivariate			
Primary outcome	OR	95% CI	*p* value	Primary outcome	OR	95% CI	*p* value
28-day mortality	1.17	0.90–1.54	0.24	28-day mortality	1.19	0.86–1.65	0.29

Secondary outcome	OR	95% CI	*p*-value	Secondary outcome	OR	95% CI	*p*-value
Severe MOF	1.04	0.74–1.48	0.82	Severe MOF	1.08	0.73–1.56	0.72
ICU Admission	1.18	0.78–1.78	0.43	ICU Admission	1.24	0.77–1.99	0.38
ED Discharge	0.62	0.46–0.82	< 0.001	ED Discharge	0.88	0.64–1.19	0.40

Multivariate regression includes adjustment for age, body mass index, cardiovascular disease, respiratory disease, upper respiratory tract and urinary site of infection. Intensive care unit admission was only assessed in patients with no limitations of care (n = 504).

*ACE2* angiotensin converting enzyme 2, *OR* odds ratio, *CI* confidence interval, *MOF* multiple organ failure, *ICU* intensive care unit, *ED* emergency department.

**Fig. 1:**
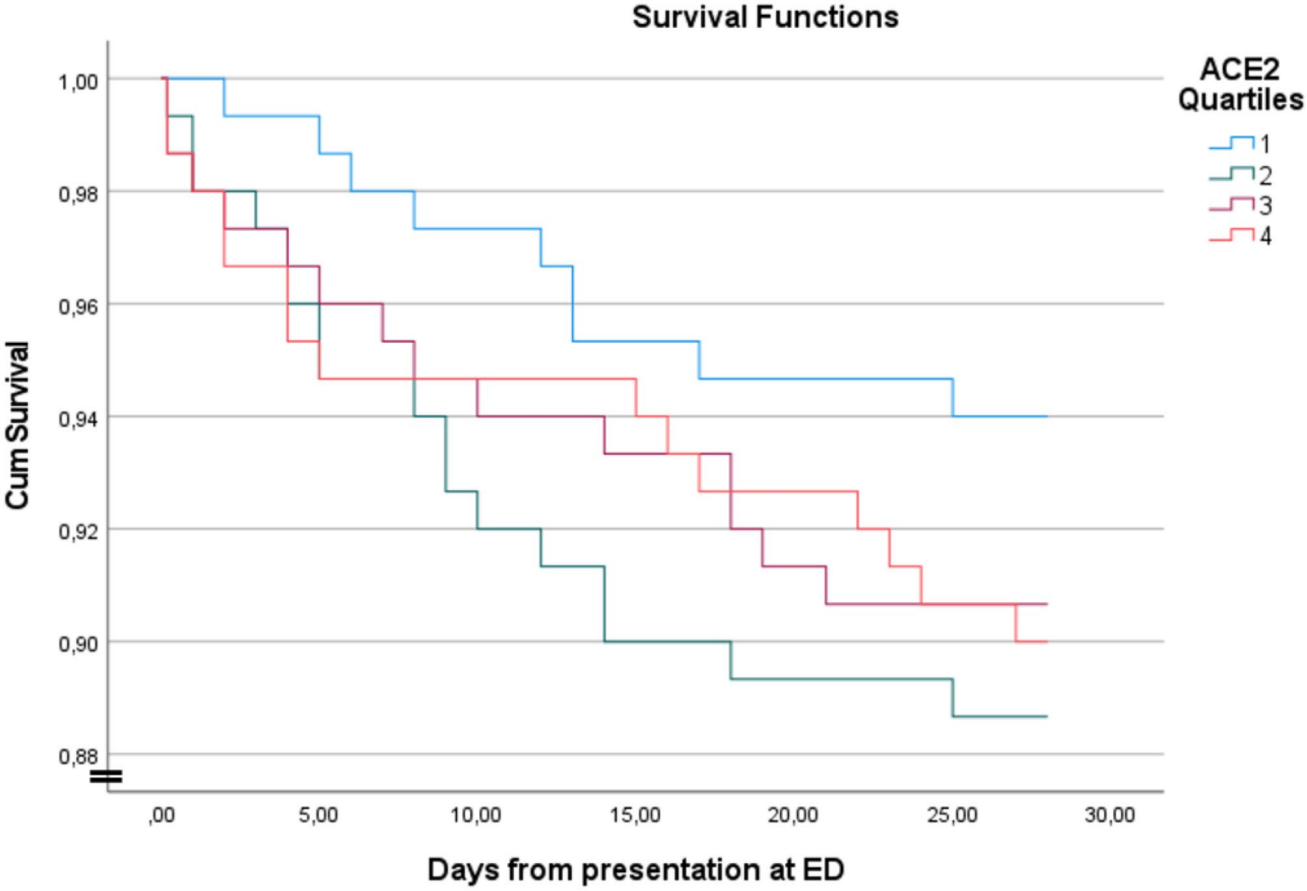
Kaplan–Meier curve of 28-day mortality in ACE2 is divided into quartiles. Quartile 1: < 2.66 ng/ml. Quartile 2: 2.66–4.64 ng/ml. Quartile 3: 4.64–9.31. Quartile 4: > 9.31 ng/ml. *P* value derived from log-rank test shows no significant pattern (*p* = 0.420). ACE2: angiotensin converting enzyme 2.

When dividing the whole populations into quartiles based on ACE2 levels, there was no apparent pattern in the Kaplan Meier plot as regards the association between ACE2 levels and 28-day mortality (Fig. 1). This was confirmed with the Log Rank comparison showing no significant trend between the quartiles and the survival curve (*P* = 0.42).

### ACE2 as a biomarker

**Fig. 2:**
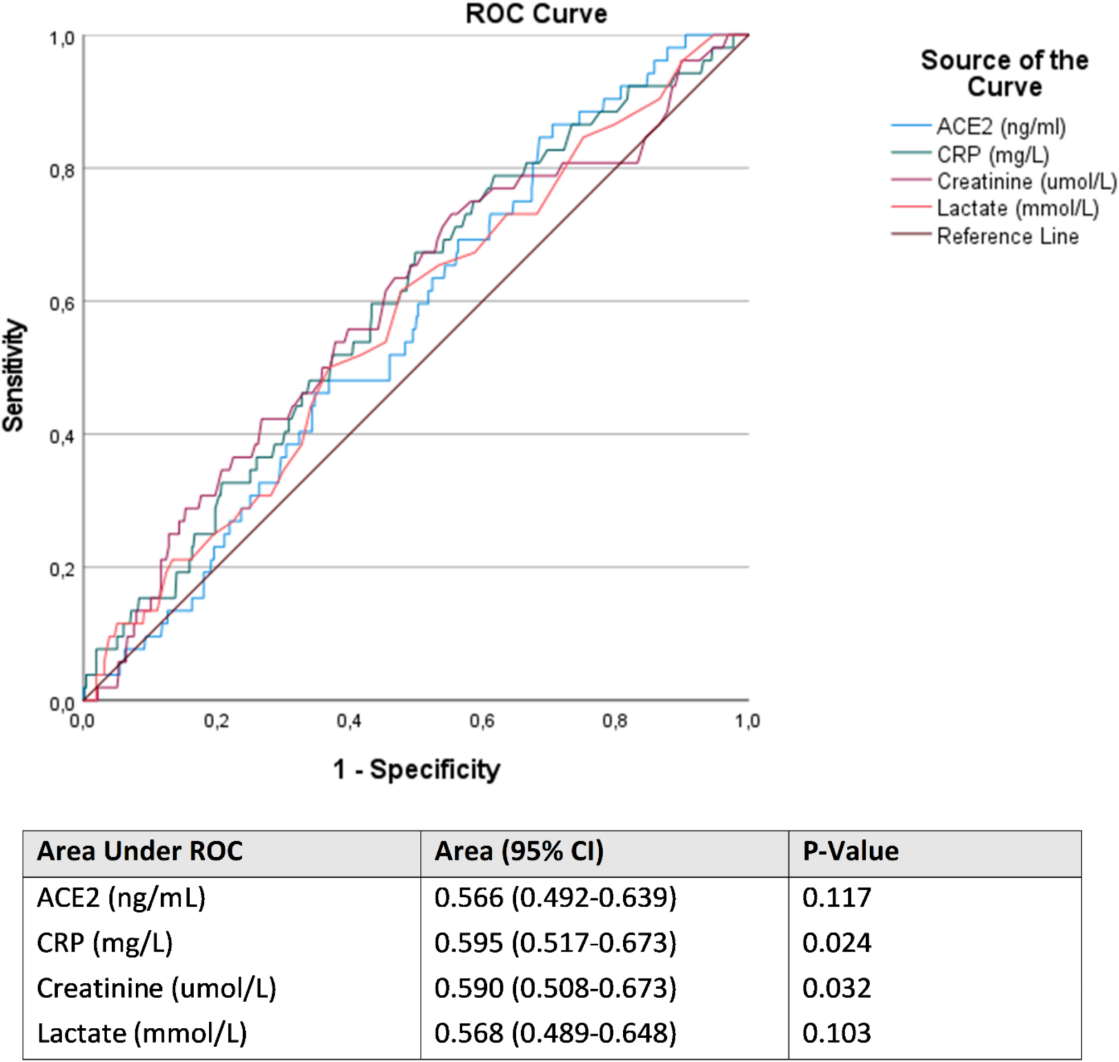
ROC curves for biomarkers ACE2, CRP, creatinine and lactate corresponding to 28-day mortality. *P*-value derived from Dunn’s test comparing the area under ROC curves. *ROC* Receiver operating characteristics, *ACE2* angiotensin converting enzyme 2; *CRP* C-reactive protein.

When assessing the sensitivity/specificity of ACE2 levels (AUC 0.55) to predict 28-day mortality, we found that other biomarkers commonly used in the clinical setting, including C-reactive protein (CRP, AUC 0.60), creatinine (AUC 0.59), and lactate (AUC 0.57) showed a stronger predictive value (Fig. 2). Similarly, vital signs registered at admission to the ER showed a stronger predictive value than plasma ACE2 levels to predict 28-day mortality (AUC 0.57–0.67).

### Association between ACE2 levels and organ failure

**Fig. 3:**
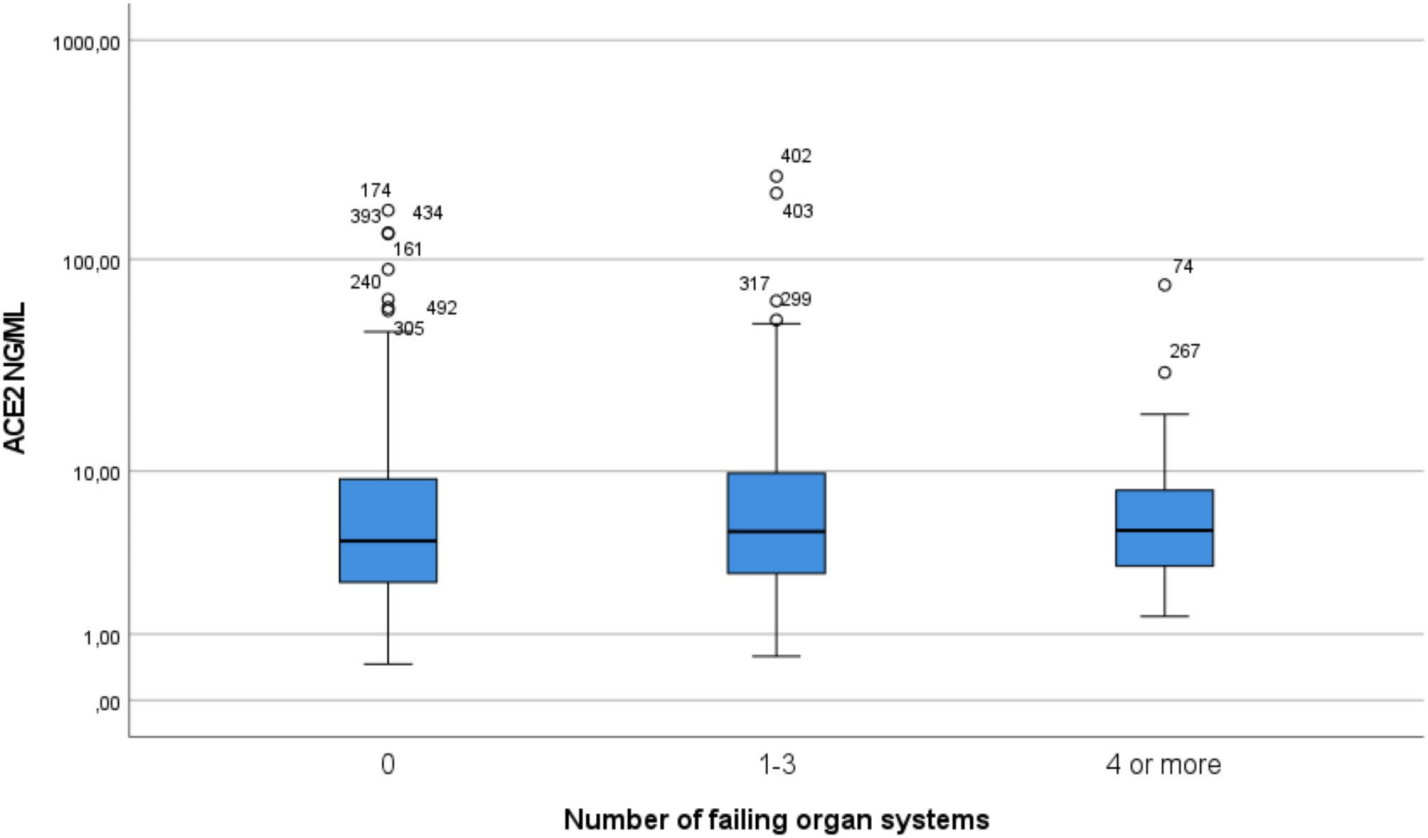
Boxplots of ACE2 levels grouped into number of failing organs. No significant differences were seen when calculated with pairwise Wilcoxon test. *ACE2* angiotensin converting enzyme 2.

There were 285 patients who did not develop organ failure, 282 patients with 1–3 failing organs and 33 patients with 4 or more organs failing (MOF). Plasma ACE2 levels were not higher in patients with MOF compared to patients who did not develop organ failure (Fig. 3). In the multivariate analysis, plasma ACE2 levels were not associated with MOF (OR 1.08 0.73–1.56. *P* = 0.72) (Table 3).

### Level of care

When assessing the association of plasma ACE2 and ED discharge, an association is observed in the univariate regression OR 0.62 (95% CI 0.46–0.82, *p* < 0.001), but following adjustment in the multivariate analysis the OR 0.88 (95% CI 0.64–1.19) is no longer significant (*p* = 0.40) (Table 3).

**Fig. 4:**
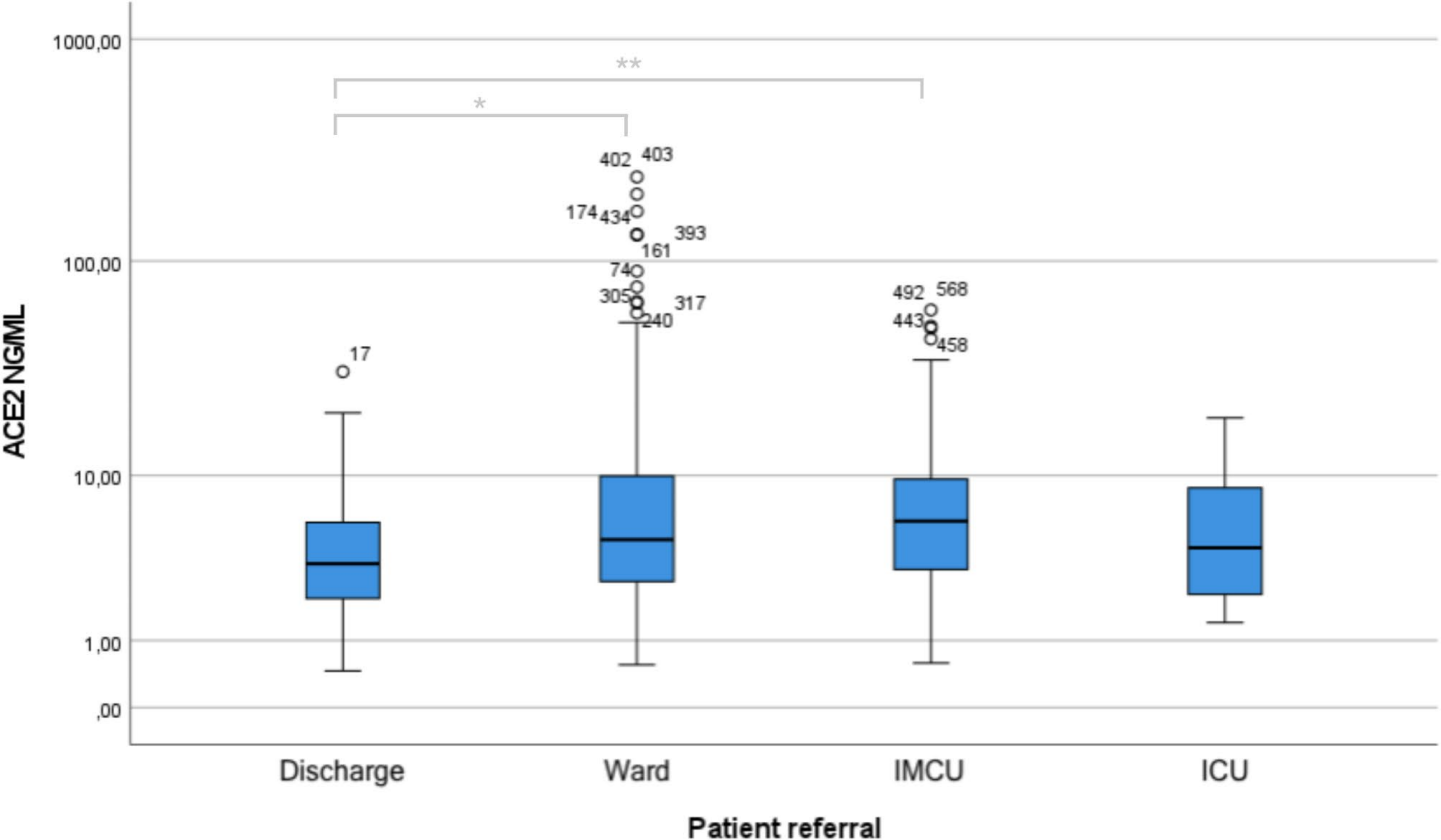
Boxplots of ACE2 levels grouped after patient referral from the emergency department. P-values derived from pairwise Wilcoxon test. *P < 0.05, **P < 0.01. No significance remained after adjustment for age, body mass index, cardiovascular disease, respiratory disease, upper respiratory tract and urinary site of infection, in a multivariate logistic regression (ward: *P* = 0.53, IMCU: *P* = 0.14, ICU: *P* = 0.90). *ACE2* angiotensin converting enzyme 2; *IMCU* intermediate care unit; *ICU* intensive care unit.

Among the 600 patients included in the analyses, 68 patients were discharged from the ED, 367 patients were treated in the regular ward, 136 patients were treated in the intermediate care unit (IMCU), 28 patients were treated in the intensive care unit (ICU), and 1 patient missed data of referral. Compared to patients that were discharged from the ED, ACE2 levels were higher in patients admitted to regular ward (*p* = 0.017) and to the IMCU (*p* = 0.004), but no significant increase in ACE2 levels was detected among those admitted to ICU (Fig. 4). The observed associations between higher plasma ACE2 levels and admission to regular ward or the IMCU were however not statistically significant when adjusting for age and comorbidities (*P* = 0.53, *P* = 0.14).

## Discussion

In this prospective, single-center, observational study, we examined plasma levels of ACE2 in patients with sepsis admitted to the ED. We found that plasma ACE2 was associated with patient comorbidity but not with 28-day mortality. In the receiver operating characteristics (ROC) analysis, ACE2 levels did not predict 28-day mortality, in contrast to the widely used laboratory biomarkers CRP, creatinine. Whereas plasma ACE2 did not predict mortality, CRP and creatinine were only weak predictors (AUC < 0.6), as were vital signs (AUC 0.57–0.67), underlining the complexity of risk prediction in sepsis patients.

To our knowledge there are no other studies investigating plasma ACE2 levels in patients with sepsis admitted to the ED. The results of the present study therefore add to the discussion of ACE2’s role in infectious diseases and as a biomarker predicting mortality^6,9,16^. In the present study, we confirm findings from other studies in the general population and cohorts of COVID-19 patients which have shown that plasma ACE2 levels increase with age, are higher in men, and in patients with hypertension, congestive heart failure, diabetes, obesity and cancer^9,17^. In relation to what has been found in studies on COVID-19 patients, we could not confirm that plasma ACE2 predicts organ failure^9,16,18^. In line with other studies^10,17,19,20^, plasma ACE2 levels reflected male sex, age and comorbidities also in the present study sample of sepsis patients. Interestingly, ACE2 has been proposed as a biomarker of frailty due to its close association with higher BMI, worse physical function and increased dependency^21^. We did not analyze frailty, but it is well accepted that frailty is associated with increased comorbidity, particularly involving chronic diseases. Thus, a partial explanation for our finding could be that plasma ACE2 in the study subjects reflects a higher level of comorbidity and frailty. We also found an association between plasma ACE2 and admission to ward and IMCU indicating an association with disease severity, although only when the analyses were not adjusted for age and comorbidities.

Although there has also been some discrepancy as regards the prognostic value of ACE2 in predicting COVID-19 outcome^16,22^, there is a strong rationale for using ACE2 as a marker in COVID-19. Given that SARS-COV-2 utilizes the membrane-bound ACE2 as a receptor and induces its shedding by activating other membrane-bound enzymes such as ADAM17 and ADAM10^4^, it is plausible that circulating ACE2 levels in COVID-19 patients to a greater extent reflects disease severity, compared to sepsis, based on the findings of the present study. Strengthening this argument, patients with COVID-19 had higher circulating ACE2 levels compared to patients with influenza and similar disease severity^18^.

In patients with sepsis, the use of ACE2 as a potential therapy is controversial as ACE2 has been suggested to represent a double-edged sword^6^. On the one hand, high ACE2 activity can protect against both sepsis-induced left ventricular dysfunction, lung inflammation, ARDS, early-stage acute kidney injury (AKI), and mortality by increasing ANG 1–7 and decreasing ANGII activity^6,23–25^. On the other hand, increased ACE/ANG II is crucial for survival in septic shock, by increasing the vascular resistance and elevating blood pressure^6^, why high ACE2 levels theoretically could increase disease severity by decreasing the natural RAS response to septic shock. Therefore, the balance of the RAS may be of great importance in care of patients exposed to sepsis and septic shock, where ACE2 activity may promote tissue protection and survival under certain conditions, but negatively impact tissue protection and survival in other conditions, by decreasing blood pressure, tissue perfusion, thereby leading to organ failure^6^. The present study does not provide evidence indicating benefits from using ACE2 as a potential therapy in patients with sepsis. It is possible that future studies investigating the longitudinal dynamics of ACE2 in sepsis patients could provide more information.

There is a strong rational for increasing the understanding of how RAS activation can be utilized in the care of sepsis patients. In studies of mice and sheep, the administration of ANG 1–7 reduces LPS/septic shock induced inflammation and attenuates organ injury^26,27^. There are concerns that ANG 1–7 administration could worsen septic shock by causing vasodilatation, however, such effects were not observed in the septic shock sheep model^27^. At the other end of the spectrum, several post-hoc studies based on the Angiotensin II for the Treatment of High Output Shock (ATHOS-3) trial have found that ANGII administration can provide potential benefit on survival on subsets of patients with vasodilatory shock^28^. Administration of supraphysiological levels of ANGII, or inhibiting ACE2 activity in swine leads to severe lung and kidney injury, reduced blood oxygenation and hypercoagulopathy^29^.

Theoretically, the timing of ANGII and/or ACE2/ANG1–7 administration to patients with sepsis could be of great importance. In the present study we only have data on ACE2 levels at admission, which does not seem sensitive enough to identify patients at risk of severe outcome. Future studies should include repeated blood sampling, evaluating the dynamics of ACE2 and RAS during disease progression, as a possible key to identifying patients at risk, and finding eventual therapeutic time windows. That the timing of potential modulation of the RAS may be crucial was further demonstrated in a recent *post-hoc* analysis showing that patients in vasodilatory shock administered ANGII when the norepinephrine-equivalent dose was ≤ 0.25 µg/kg/min had higher survival rates compared to placebo or when the norepinephrine-equivalent dose was > 0.25^28^.

### Strengths and limitations

One important strength of the present prospective observational cohort study is the large study sample. Also, all patient records in this study were thoroughly revised by infectious disease physicians to assure correct diagnoses. This study, however, has several limitations. Patients were only enrolled during office hours, implicating potential selection bias. Also, only admission samples were available, which excludes analyses of dynamic changes. Studies on severe COVID-19 patients indicate that a dramatic increase in circulating ACE2 levels is observed first a few days after admission. Reindl-Schwaighofer et al.^18^ found that circulating ACE2 levels in patients with severe COVID-19 increased sevenfold from early to late following admission, and that this increase was accompanied by increased ANG1–7 levels and ANG1–7/ANGII ratio, suggesting a shift towards the alternative RAS and increased organ protection later in the disease course. Finally, no healthy controls were used, why we cannot say anything about how the plasma ACE2 levels in sepsis patients relate to the general population. The study data was gathered when the Sepsis-2 criteria defined sepsis^30^, which is no longer the current definition.

In the present study, we included all patients presenting with symptoms reaching the sepsis-2 definition. This could be seen as a limitation since an unselected sample of sepsis patients were included. The site of infection underlying sepsis could be more or less predictive of mortality. However, upon admission to the ED, the underlying cause of sepsis is not always clear. To account for differences in the underlying cause of sepsis, we included infection site in the multivariable analyses when significant differences were observed between survivors and non-survivors.

In conclusion, in patients with sepsis, plasma ACE2 levels are increased in those with male sex, high age and comorbidities, including diabetes, obesity, cardiovascular and cancer diseases. ACE2 levels were not associated with 28-mortality, nor with organ failure. Contrary to our hypothesis and to animal studies, we did not find ACE2 to be a promising marker in sepsis. To our knowledge, this is one of the first studies elucidating on plasma ACE2 in patients with sepsis admitted to the ED. Future studies which investigate the dynamics of circulating ACE2 levels in longitudinal studies with repeated sampling are warranted. An increased understanding of the dynamics of RAS dysfunction in sepsis patients could potentially help generate individualized treatment options, especially in those patients who progress into vasodilatory shock.

## Patients and methods

### Study design and setting

This prospective single-center, observational cohort study was described in detail previously^31^. It was conducted at Skåne University Hospital’s ED in Malmö, Sweden, a department that serves around 400,000 individuals and there are some 85,000 emergency visits annually. Written informed consent was obtained from all the participants and/or their legal guardians. For patients with reduced consciousness at inclusion, written informed consent was obtained retrospectively. The study design and consent process were approved by the Regional Ethical Board in Lund (DNR: 2013/635). We applied the STROBE guidelines^32^. All methods were performed in accordance with the relevant guidelines and regulations.

### Participants

From December 2013 to February 2015, adults seeking ED care on weekdays (6 AM to 6 PM) were screened by research nurses. Inclusion criteria, following the sepsis-2 definition^30^, required suspected infection plus ≥ 2 systemic inflammatory response syndrome (SIRS) criteria: (1) Body temperature < 36 °C or > 38 °C, or self-reported fever/chills within 24 h before ED visit, (2) Respiratory rate > 20 breaths/min, (3) Heart rate > 90 beats/min. White blood cell count was not included due to unavailability during screening.

The study did not have a predetermined sample size and instead included patients on a convenience basis throughout the study duration.

### Variables

The primary outcome was 28-day mortality, with secondary outcomes including organ system failure count, ICU admission, and ED discharge. Organ system failure was categorized as: (1) no failure, (2) intermediate failure (one to three systems), and (3) multiple organ failure (MOF) (four or more systems) within 48 h of ED arrival. ICU admissions were tracked throughout the follow-up period. Intensive care unit admission was only assessed in patients with no limitations of care. Level of care depends on the patient’s treatment needs. A general ward provides oxygen (10–15L), IV medication, and basic care. IMCU admission is required for non-invasive ventilation (NIV) or inotropic support. ICU admission is needed for ventilator support. Premorbid comorbidities were recorded and classified, as detailed in Table 1.

Organ system failure is defined by the criteria as follows. Central Nervous System: Confusion, drowsiness, or loss of consciousness. Circulatory Failure: Systolic blood pressure < 90 mmHg, mean arterial pressure (MAP) < 70 mmHg, a decrease in systolic blood pressure > 40 mmHg, or the requirement for vasopressors to maintain blood pressure. Respiratory Failure: Arterial oxygen saturation (SaO₂) < 90% or the need for mechanical ventilation. Kidney Failure: An increase in serum creatinine > 44 μmol/L between any two measurements, a requirement for acute renal replacement therapy, or a 1.5-fold increase in baseline creatinine with an initial value > 160 μmol/L within 48 h. Liver Failure: Total serum bilirubin > 40 μmol/L. Hematologic Dysfunction: Platelet count < 100 × 10⁹/L, international normalized ratio (INR) > 1.5, or activated partial thromboplastin time (aPTT) > 60 s. Metabolic Dysfunction: Serum lactate > 3.5 mmol/L^31^.

### Data sources

We collected patient demographics, comorbidities, infection site, and hospital ward type through systematic review of medical records, overseen by infectious disease physicians.

### Biomarkers

Blood samples were taken from peripheral veins within an hour of ED admission. All biomarkers, except ACE2, underwent standard analysis at the certified hospital laboratory in Malmö. ACE2 analysis involved freezing ethylenediaminetetraacetic acid plasma samples within two hours and storing them at -80˚C for subsequent batch analysis. Plasma concentration of ACE2 measurement were conducted at Department of Clinical Chemistry and Pharmacology, Academic Hospital, Uppsala, Sweden with sandwich enzyme‐linked immunosorbent assays (ELISA) retrieved from Adipogen Life Sciences, “ACE2 human ELISA kit” (catalog number AG‐45B‐0023‐KI01; Adipogen Life Sciences) According to the manufacturer, the intra-assay and inter-assay coefficient of variation ranged from 2 to 5% [https://adipogen.com/ag-45b-0023-ace2-human-elisa-kit.html/].

### Statistics

Continuous variables were tested if normally distributed or not with Shapiro Wilks, where *p* > 0.05 was considered normally distributed. When comparing groups based on continuous variables, the non-normally distributed variables were examined with Mann–Whitney U test for two groups and reported medians along with their respective interquartile ranges (IQR), while normally distributed variables were examined with t-test and presented with 95% confidence interval (CI). When comparing more than two groups, we employed the Kruskal–Wallis´ rank sum test. If this test indicated significant differences between the groups, we proceeded to perform Dunn’s test for pairwise comparisons. To evaluate disparities in proportions, we utilized Pearson’s chi-squared test.

To analyze outcomes, we used uni- and multi-variable binary logistic regression. The results of the regression analyses were presented as odds ratios (OR) with corresponding 95% confidence intervals (CI). The OR for ACE2 was calculated per standard deviation (SD). Body mass index (BMI) was categorized into underweight (< 18.5 kg/m^2^), normal (18.5–25), overweight (25–30), and obese (> 30). If a parameter needed transformation due to skewness, we applied the base 2 logarithm. We divided ACE2 levels into quartiles and assessed the difference in Kaplan–Meier curves using the log-rank test. We utilized IBM SPSS Statistics version 28.0.1.0 (142) as our statistical software.

Covariables in the multivariate regression analyses were set to the variables correlating with 28-day mortality as seen in Table 1. The covariables adjusted for were age, body mass index (BMI), cardiovascular disease (CVD), respiratory disease, upper tract respiratory infection (UTRI) and urinary infection (UTI). A *p*-value < 0.05 was considered significant for all hypothesis tests.

## Data Availability

Upon reasonable request, all data is available from corresponding author FF.
